# Implementation of a seven-day hospitalist program to improve the outcomes of the weekend admission: A retrospective before-after study in Taiwan

**DOI:** 10.1371/journal.pone.0194833

**Published:** 2018-03-26

**Authors:** Nin-Chieh Hsu, Chun-Che Huang, Chin-Chung Shu, Ming-Chin Yang

**Affiliations:** 1 Division of Hospital Medicine, Department of Traumatology, National Taiwan University Hospital, Taipei, Taiwan; 2 Department of Internal Medicine, National Taiwan University Hospital, Taipei, Taiwan; 3 Department of Family Medicine, Kaohsiung Medical University Hospital, Kaohsiung Medical University, Kaohsiung, Taiwan; 4 Institute of Health Policy and Management, College of Public Health, National Taiwan University, Taipei, Taiwan; VU university medical center, NETHERLANDS

## Abstract

**Objective:**

Patients admitted during weekends may have worse outcomes than those during weekdays. Adjusting the practice of senior physicians over weekends may reduce the weekend effect.

**Design:**

A controlled before-after study, with propensity score matching (PSM) for potential confounding variables, to compare outcomes between weekday and weekend admissions.

**Setting:**

A 2000-bed medical centre in Taiwan

**Participants:**

Hospitalised general medicine patients cared for by traditional internal medicine teams (pre-intervention cohort) and those cared for by hospitalists after introducing a seven-day hospitalist program in the first six-month (post-intervention cohort) and following three-year periods.

**Main outcome measures:**

Proportion of intensive care unit (ICU) admissions, cardiopulmonary resuscitation (CPR) events, and in-hospital mortality.

**Results:**

The pre-intervention cohort included 982 patients. Significantly higher mortality rates (11.3% vs. 6.2%, *p* = 0.032) were recorded in the case of weekend admissions, with similar proportions of ICU admission and CPR events. The post-intervention cohort included 601 patients. No significant difference was recorded in any of the main outcomes between weekday and weekend admissions. PSM for pre-intervention and post-intervention cohort showed shorter LOS after intervention, with no difference in ICU admission, CPR, and morality for the weekday and weekend admissions, respectively. The three-year cohort that followed, consisting of 3315 patients, showed no difference of outcomes between weekday and weekend admissions. After PSM, there were no significant differences in ICU admission rates (1.0% vs. 1.8%), CPR (0.3% vs. 0.2%) events and hospital mortality rates (8.1% vs. 8.5%), when weekday and weekend admissions were compared.

**Conclusions:**

The seven-day hospitalist program shows potential in providing equally safe care for both weekday and weekend general medicine admissions with sustainable development.

## Introduction

Several studies have reported that higher mortality rates were observed in patients admitted to hospitals on weekends, especially in the case of emergency admissions [1–3]. Possible explanations for this ‘weekend effect’ may include differences in staffing [4], the unavailability of important procedures [5] and variations in the patient cohorts [6]. It has been expected that focussing on the working practices of senior hospital doctors at the weekends could improve care quality and reduce the weekend effect [7].

Over the past two decades, the hospitalist system has become the dominant inpatient care model in the United States [8, 9]. Numerous studies have demonstrated the advantages of using hospitalists to care for general medical [10, 11], paediatric [12], stroke [13] and surgical patients [14, 15]. Potential advantages of the hospitalist system include greater expertise in inpatient care and better availability of physicians during the duration of hospitalisation [16]. It has been hypothesised that weekend effect results from different staffing between weekdays and weekends [4], but hospitalists are typically available at weekends. Therefore, introducing hospitalists into the traditional inpatient care model can potentially provide equal quality of care during weekends because of the typical 24-hours/7-day coverage [17].

In late 2009, Taiwan established a pioneer hospitalist program for acute general medicine admissions, which produced similar outcomes of care. Higher efficiency in throughput was observed, when compared to traditional internal medicine practices [18]. However, improvements in quality indicators and outcomes have been inconsistent [16, 19]. Current literature does not determine whether the hospitalist system can waive from the weekend effect. The research hypothesis was that introducing a hospitalist system would make consistent outcomes for both weekday and weekend admissions. Therefore, this study aimed to investigate the outcomes of general medicine hospitalisations before and after introducing a 7-day hospitalist system.

## Materials and methods

### Study setting

The study was conducted at a 2000-bed medical centre in North Taiwan. The idea of the hospitalist program was conceived in October 2009. As part of this, a pioneering, hospitalist-run acute general medicine unit had both attending physicians and nurse practitioners (NPs) admitting general medicine patients from the emergency department (ED). The outcomes of the first three months were reported [19]. In contrast to traditional academic medical services, the hospitalist program did not have resident physicians to take care of inpatients, except during the night shift, when there was resident physician-coverage under hospitalist supervision.

In January 2010, three shifts were designed for the hospitalist team, for the weekdays. New admissions from the ED, which typically presented between 11 am and 5 pm, were assigned to both the day shift (8 am to 5 pm) and the bridge shift (1 pm to 11 pm) hospitalists. The night shift was from 11 pm to 8 am; the hospitalist on duty took handoffs from the bridge shift and covered a maximum of 36 beds over the course of the night. During the weekends, one hospitalist acted as the on-duty physician to both cover a 24-hour shift and admit new patients. The remaining hospitalists who had daytime duty made the rounds of their patients to maintain continuous care, and to also ensure that all inpatients could approach their daytime attending hospitalists at any time during their stay. The role of nurse practitioners (NPs) in the hospitalist program was to collaborate with hospitalists to provide care to hospitalised patients. NPs could help hospitalists to evaluate the patients’ conditions, arrange examinations that should be performed outside the ward, contact the consultants and document records. They could also perform nursing procedures including wound dressing, and nasogastric tube and urinary catheter insertions. The hospitalist ward run 7 days per week, and the workload was not significantly low during weekends. This is why we needed to maintain the same staffing of NPs during weekends. The staffing structure of the hospitalist program, on both weekdays and weekends, was presented in comparison with that of the traditional general medicine (Table 1) structure. The patient-to-nurse ratio, consultation service, and social supporting system were the same in these two systems.

**Table 1:** Comparison the settings of the traditional general medicine and hospitalist staffing on weekdays and weekends.

	Traditional general medicine staffing		Hospitalist staffing	
	Weekdays	Weekends	Weekdays	Weekends
Beds, per ward	36	36	36	36
Clinical team structure	3AP, 1CR, 3JR	3AP, 1CR, 3JR	3AP, 4NP	3AP, 4NP
Daytime manpower	3AP, 3JR	1JR, 1CR	3AP, 4NP	1AP, 4NP
Night shift (11pm-8am)	1JR, 1AP	1JR, 1CR	1JR, 1AP	1JR, 1AP
AP for bedside round	3	1	3	3
AP for new admission	3	1	3	1
AP for emergency calls	3	1	3	1

Abbreviations: AP, attending physician; CR, chief resident; JR, junior resident; NP, nurse practitioner.

### Data collection

This study was based on a longitudinal hospitalist performance research study approved by the Research Ethical Committee of the National Taiwan University Hospital (NTUH, 201112161RIC). The patient data, before July 2011, was retrospectively collected from medical records. Since then, each admission to the hospitalist general medicine unit was prospectively collected. There were only missing data of the laboratory results because some tests were not mandatory for each patient on admission. Therefore, laboratory data were used descriptively without comparison. The study design and methods comparing weekday and weekend admissions were approved by the Research Ethical Committee of the NTUH (201308025RINA), which waived the requirement for informed consent. Written consent from study objects was not obtained and confidentiality assurances were addressed by abiding to data regulations of the NTUH.

Weekend admission was defined as an admission between 12:00 am on Saturday and 11:59 pm on Sunday. Patients admitted during the remaining time periods were considered weekday admissions [4]. The baseline characteristics of each patient were recorded, including age, gender, admission Barthel index (BI) and admission diagnosis. The BI was used to rate the patients’ activities of daily living on admission [20]. Since the study used primary patient-level data, laboratory data on admissions was also compared between groups to evaluate disease severity.

### Measurements

The primary outcome variables were the length of hospital stay (LOS), proportion of intensive care unit (ICU) admissions, cardiopulmonary resuscitation (CPR) events and hospital mortality rates.

The pre-intervention group consisted of a comparable patient group cared for by traditional internal medicine models, just before the implementation of the hospitalist system. From November 1, 2009 to December 31, 2009, patients admitted from the ED to the seven traditional general medicine wards (with a total of 239 beds) were sampled. The medical records of the patients were retrospectively reviewed.

The post-intervention group included all the patients admitted to the then-new hospitalist-care ward from January 2010 to June 2010. Baseline characteristics, hospital course and outcomes were analysed, in both the pre and post-intervention cohorts, following the same steps. These analyses focused on the differences between weekday and weekend admissions, rather than on the absolute value of each outcome. To evaluate the long-term effect of intervention, a three-year cohort- from January 2010 to December 2012- was also collected for analysis. By using medical records of hospitalisation, there was no missing data or loss to follow-up.

### Statistical analysis

Data was analysed using the SPSS software (version 16.0, SPSS Inc., Chicago, IL, USA). Inter-group differences were compared using the independent *t*-test for continuous variables; categorical variables were compared using the Fisher’s exact test. Acute medical admissions, unlike either elective surgery or scheduled admissions, occurred on both weekdays and weekends. Because of the inherent differences between weekday and weekend admissions, in terms of baseline characteristics, we used the propensity score matching (PSM) with a ratio of 1:1 to adjust these differences. Propensity scores were estimated using a logistic regression model of the patient demographics and clinical characteristics in the three-year follow-up cohort. The covariate balance between the matched groups was examined. Descriptive analyses were repeated to compare outcome variables. Because of non-parallel comparison, outcomes of weekday and weekend admissions were also compared between pre- and post-interventions using the PSM technique. The statistical significance was set at a two-sided *p*<0.05.

## Results

### Main outcomes of the pre- and post-intervention groups

From November 1, 2009 to December 31, 2009, a total of 982 patients admitted from the ED were enrolled. The clinical courses between weekday and weekend admissions showed that the LOS was similar. The proportion of ICU admissions (1.4% vs. 1.2%) and CPR events (1.4% vs. 0.6%) during hospitalisation were slightly higher in weekend admissions, but were statistically insignificant. However, the crude rate of hospital mortality was significantly higher in weekend admissions compared to weekday admissions (11.3% vs. 6.2%, *p* = 0.032) (Table 2).

**Table 2:** Characteristics and outcomes of the pre- and post-intervention cohort in caring for weekday and weekend admissions.

	Pre-intervention		*p*	Post-intervention		*p*
	Weekday admission (n = 840)	Weekend admission (n = 142)		Weekday admission (n = 430)	Weekend admission (n = 171)	
Age (yr)	65.8 ± 18.1	66.9 ± 16.9	0.517	69.7 ± 15.9	69.4 ± 15.8	0.849
Male sex	449 (53.4)	81 (57.0)	0.467	202 (47.0)	82 (48.0)	0.857
BI at admission	66.7 ± 35.0	62.8 ± 35.8	0.223	44.1 ± 36.8	48.6 ± 37.0	0.425
Admission diagnosis						
Pneumonia	220 (26.2)	40 (28.2)	0.608	33 (7.7)	16 (9.4)	0.510
Urinary tract infection	181 (21.5)	33 (23.2)	0.660	31 (7.2)	11 (6.4)	0.860
Intra-abdominal infection	80 (9.5)	14 (9.9)	0.878	12 (2.8)	7 (4.1)	0.440
Gastrointestinal bleeding	88 (10.5)	17 (12.0)	0.559	7 (1.6)	7 (4.1)	0.080
Renal failure	55 (6.5)	6 (4.3)	0.351	7 (1.6)	3 (1.8)	1.000
Exacerbation of COPD	40 (4.8)	9 (6.3)	0.406	5 (1.2)	3 (1.8)	0.694
Congestive heart failure	25 (3.0)	4 (2.8)	1.000	10 (2.3)	3 (1.8)	1.000
Laboratory on admission						
WBC	11.1 ± 6.8	11.0 ± 6.9	0.827	9.8 ± 5.3	10.6 ± 5.1	0.334
Hemoglobin	12.3 ± 7.9	11.6 ± 24	0.318	11.1 ± 2.4	10.9 ± 2.6	0.633
Albumin	3.5 ± 0.7	3.3 ± 0.9	0.119	3.1 ± 0.8	3.6 ± 0.6	0.010
BUN	26.6 ± 22.5	26.2 ± 19.0	0.851	34.8 ± 32.9	35.8 ± 29.5	0.847
CRP	6.5 ± 6.9	8.1 ± 6.6	0.092	6.1 ± 5.7	8.4 ± 9.6	0.237
Course and outcomes						
Hospital LOS (days)	13.2 ± 14.6	13.2 ± 19.6	0.967	8.9 ± 7.7	9.5 ± 9.5	0.440
ICU admission	10 (1.2)	2 (1.4)	0.688	27 (6.3)	4 (2.3)	0.064
CPR event	5 (0.6)	2 (1.4)	0.287	4 (0.9)	0 (0)	0.582
Hospital mortality	52 (6.2)	16 (11.3)	0.032	39 (9.1)	17 (9.9)	0.757

Data are expressed as mean ± standard deviation or number of cases (%).

Abbreviations: BI, Barthel index; BUN, blood urea nitrogen; COPD, chronic obstructive pulmonary disease; CPR, cardiopulmonary resuscitation; CRP, c-reactive protein; ICU, intensive care unit; LOS, length of stay; WBC, white blood cells.

The post-intervention, six-month cohort included 601 patients. A similar LOS was recorded in both weekday and weekend admissions. In addition, there was no significant difference in ICU admission rates, CPR events and hospital mortality rates (Table 2). However, the proportion of ICU admissions was higher among weekday-admitted patients after the introduction of hospitalist program. This condition may be related to the difference in BIscores of both weekday and weekend admissions between the pre-intervention and post-intervention groups. Our results given in Table 2 showed that the post-intervention group had lower mean scores of Barthel index on both weekday and weekend admissions (44.1 and 48.6, respectively) compared to the pre-intervention group (66.7 and 62.8, respectively).

PSM for the pre-intervention and post-intervention group showed comparable demographics, clinical characteristics and laboratory data after PSM. ICU admission, CPR events and hospital mortality showed no statistical difference for both weekday and weekend admissions. However, LOS were significant shorter in post-intervention group, both for weekday admissions (15.4 vs. 8.8 days, p<0.001) and weekend admissions (10.3 vs. 12.3 days, p = 0.037) (Table 3).

**Table 3:** Results of propensity score matching for care on weekday and weekend admissions between the pre- and post-intervention cohort.

	Weekday admission		*p*	Weekend admission		*p*
	Pre-intervention (n = 353)	Post-intervention (n = 353)		Pre-intervention (n = 90)	Post-intervention (n = 90)	
Age (yr)	70.1 ± 16.5	68.4 ± 16.2	0.081	66.9 ± 16.5	65.6 ± 16.5	0.600
Male sex	185 (52.4)	168 (47.6)	0.228	54 (60.0)	45 (50.0)	0.231
BI at admssion	49.7 ± 34.8	50.9 ± 37.0	0.448	60.7 ± 38.0	62.8 ± 36.8	0.943
Admission diagnosis						
Pneumonia	88 (24.9)	81 (23.0)	0.597	20 (22.2)	18 (20.0)	0.855
Urinary tract infection	67 (19.0)	57 (16.2)	0.373	10 (11.1)	8 (8.9)	0.805
Intra-abdominal infection	8 (2.3)	9 (2.6)	1.000	1 (1.1)	1 (1.1)	1.000
Gastrointestinal bleeding	17 (4.8)	16 (4.5)	1.000	6 (6.7)	6 (6.7)	1.000
Renal failure	14 (4.0)	16 (4.5)	0.852	3 (3.4)	2 (2.2)	0.682
Exacerbation of COPD	19 (5.4)	12 (3.4)	0.270	7 (7.8)	2 (2.2)	0.169
Congestive heart failure	12 (3.4)	16 (4.5)	0.564	2 (2.2)	3 (3.3)	1.000
Laboratory on admission						
WBC	13.2 ± 6.3	10.8 ± 5.3	0.327	9.4 ± 5.1	10.2 ± 4.8	0.534
Hemoglobin	12.2 ± 7.5	11.6 ± 2.4	0.638	11.6 ± 2.4	10.9 ± 2.4	0.163
Albumin	3.5 ± 0.7	3.9 ± 0.9	0.204	3.3 ± 0.9	3.7 ± 0.6	0.488
BUN	26.9 ± 21.2	33.1 ± 32.4	0.066	26.6 ± 19.7	21.9 ± 20.9	0.797
CRP	7.6 ± 7.5	6.3 ± 5.7	0.558	7.1 ± 6.2	8.2 ± 9.1	0.137
Course and outcomes						
Hospital LOS (days)	15.4 ± 17.4	8.8 ± 6.9	<0.001	12.3 ± 17.0	10.3 ± 11.8	0.037
ICU admission	6 (1.7)	3 (0.8)	0.588	1 (1.1)	0 (0.0)	1.000
CPR event	2 (0.6)	0 (0.0)	0.499	1 (1.1)	0 (0.0)	1.000
Hospital mortality	31 (8.8)	40 (11.3)	0.317	10 (11.1)	8 (8.9)	0.805

Data are expressed as mean ± standard deviation or number of cases (%).

Abbreviations: BI, Barthel index; BUN, blood urea nitrogen; COPD, chronic obstructive pulmonary disease; CPR, cardiopulmonary resuscitation; CRP, c-reactive protein; ICU, intensive care unit; LOS, length of stay; WBC, white blood cells.

### Evaluation of the long-term effect of intervention

**Fig 1:**
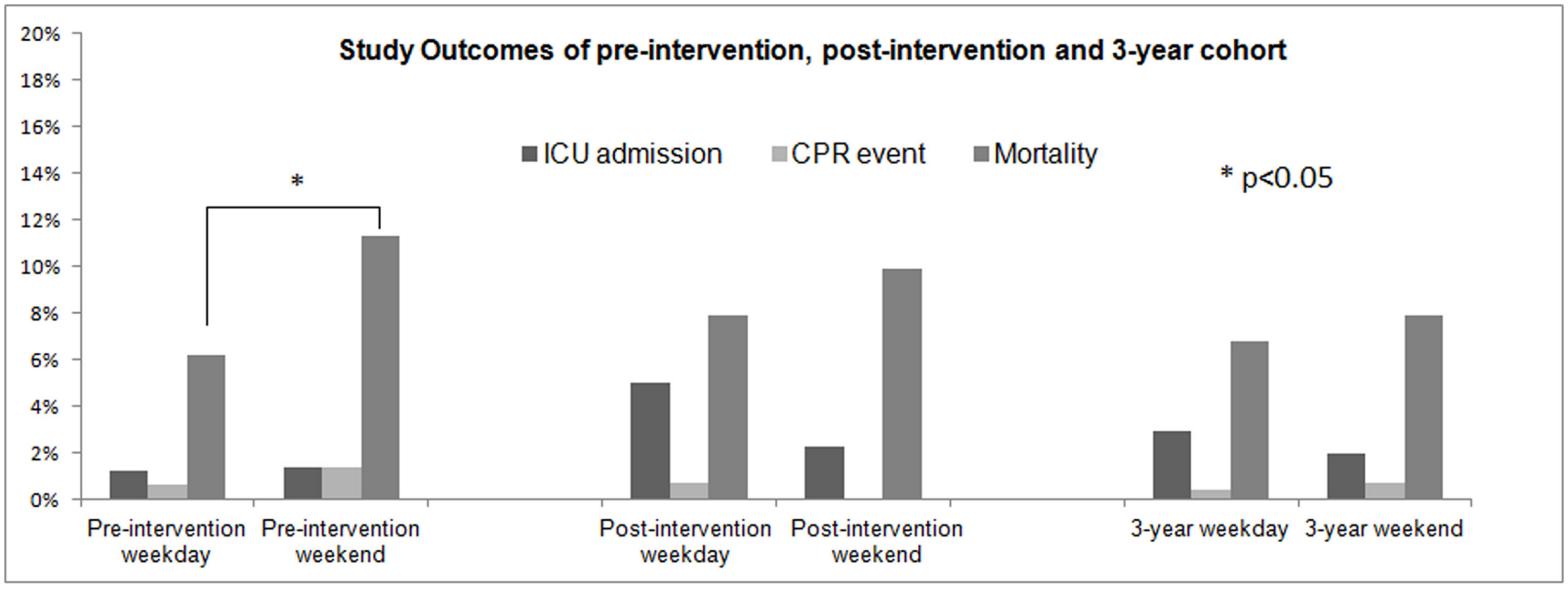
Study outcomes of pre-intervention, post-intervention and three-year follow-up cohort showed improved weekend versus weekday admission outcomes after intervention.

A total of 3315 patients, from January 1, 2010 to December 31, 2012, were admitted to the hospitalist-care ward. There was no difference between the two groups, with regards to the LOS (10.4 vs. 10.9, *p* = 0.122), proportion of ICU admission (2.9% vs. 2.0%, *p* = 0.156), CPR (0.4% vs. 0.7%, *p* = 0.259) events and hospital mortality rates (6.8% vs. 7.9%, *p* = 0.283) (Table 4). Fig 1 depicts the ICU admission, CPR event and hospital mortality rates for the pre-intervention, post-intervention and three-year cohorts, respectively.

**Table 4:** Clinical characteristics and outcomes of the three-year follow-up cohort with comparisons between weekday and weekend admissions.

	Weekday admission (n = 2417)	Weekend admission (n = 898)	*p*
Age (yr)	69.8 ± 16.3	70.4 ± 16.0	0.381
Male gender	1268 (52.5)	461 (51.3)	0.564
BI at admission	52.9 ± 41.0	48.8 ± 36.8	0.013
Diagnosis			
Pneumonia	666 (27.6)	280 (31.2)	0.040
Urinary tract infection	428 (17.7)	143 (15.9)	0.227
Intra-abdominal infection	245 (10.1)	102 (11.4)	0.307
Gastrointestinal bleeding	195 (8.1)	68 (7.6)	0.639
Renal failure	122 (5.0)	32 (3.6)	0.071
Exacerbation of COPD	86 (3.6)	25 (2.8)	0.271
Congestive heart failure	109 (4.5)	34 (3.8)	0.362
Laboratory data (admission)			
WBC	10.6 ± 7.4	11.0 ± 8.8	0.170
Hemoglobin	11.2 ± 3.0	11.2 ± 5.6	0.893
Albumin	3.5 ± 6.4	3.3 ± 2.2	0.514
BUN	34.7 ± 33.0	34.7 ± 35.8	0.986
CRP	7.7 ± 8.3	8.3 ± 10.4	0.415
Hospital course and outcome			
Hospital LOS (days)	10.4 ± 9.7	10.9 ± 9.6	0.122
ICU admission	70 (2.9)	18 (2.0)	0.156
CPR event	9 (0.4)	6 (0.7)	0.259
Hospital mortality	165 (6.8)	71 (7.9)	0.283

Data are expressed as mean ± standard deviation or number of cases (%).

Abbreviations: BI, Barthel index; BUN, blood urea nitrogen; COPD, chronic obstructive pulmonary disease; CPR, cardiopulmonary resuscitation; CRP, C-reactive protein; ICU, intensive care unit; LOS, length of stay; WBC, white blood cells.

As shown by clinical characteristics (Table 4), weekend admissions had a lower BI when compared to weekday admissions. Because of different baseline characteristics and the diagnosis distribution between weekday and weekend admissions, the PSM technique was used to allow for a fair comparison. Age, gender, and BI at admission were entered into the logistic model to generate the propensity score. After PSMwas done, both the matched 496 weekday and 496 weekend admissions showed similar patient demographics and clinical characteristics (Table 5).

**Table 5:** Results of propensity score matching for weekday and weekend admissions in the hospitalist-care cohort.

	Weekday admission (n = 496)	Weekend admission (n = 496)	*p*
Age (yr)	70.1 ± 15.6	70.0 ± 15.8	0.93
Male sex	249 (50.2)	251 (50.6)	0.899
BI at admssion	53.9 ± 36.9	51.3 ± 36.5	0.276
Diagnosis			
Pneumonia	147 (29.6)	171 (34.5)	0.118
Urinary tract infection	75 (15.1)	75 (15.1)	1.000
Intra-abdominal infection	17 (3.4)	25 (5.0)	0.27
Gastrointestinal bleeding	53 (10.7)	39 (7.9)	0.154
Renal failure	29 (5.8)	20 (4.0)	0.187
Exacerbation of COPD	27 (5.4)	16 (3.2)	0.118
Congestive heart failure	21 (4.2)	22 (4.4)	0.876
Laboratory data (admission)			
WBC	10.4 ± 6.3	10.5 ± 6.6	0.697
Hemoglobin	11.0 ± 2.6	11.3 ± 6.9	0.340
Albumin	3.2 ± 0.7	3.3 ± 2.8	0.5
BUN	33.6 ± 32.9	34.4 ± 38.2	0.716
CRP	8.1 ± 9.2	9.0 ± 12.0	0.481
Outcomes			
Hospital LOS (days)	9.9 ± 7.5	11.3 ± 10.4	0.013
ICU admission	9 (1.8)	5 (1.0)	0.299
CPR event	1 (0.2)	3 (0.3)	0.374
Hospital mortality	42 (8.5)	40 (8.1)	0.818

Data are expressed as mean ± standard deviation or number of cases (%)

Abbreviations: BI, Barthel index; BUN, blood urea nitrogen; COPD, chronic obstructive pulmonary disease; CPR, cardio-pulmonary resuscitation; CRP, c-reactive protein; ICU, intensive care unit; LOS, length of stay; WBC, white blood cell.

The clinical course and outcome indicators of the two matched groups were compared (Table 5). There were no significant differences in the ICU admission rates (1.1% vs. 1.8%), CPR events (0.3% vs. 0.2%) and hospital mortality rates (8.1% vs. 8.5%) in the cases of weekend and weekday admissions. Compared to the crude rates, the differences of the indicators were even closer after matching propensity scores. However, the mean hospital LOS was 1.4 days longer in weekend admissions when compared to weekday admissions (*p* = 0.013).

## Discussion

This study demonstrates the performance of a newly implemented hospitalist program on weekend general medicine admissions in Taiwan. No statistical difference was found between weekday and weekend admissions in the pre- and post-intervention groups apart from a shorter LOS in the post-intervention group. In addition, the hospitalist-care ward showed no difference in crude rates of hospital mortality, ICU admission and CPR events between weekday and weekend admissions. These effects could be sustained during a three-year follow-up after the implementation of the 7-day hospitalist program, even after PSM analysis.

Initially, hospitalists reported cost savings as a result of uncompromised patient outcomes [18]. The performance of the 24-hour/7-day hospitalist program during weekends is a reflection of the hospitalists’ effort and value. The reason hospitalists maintain outcomes of weekend admissions are most probably the inherent around-the-clock coverage feature. In the past, a hospitalist has varyingly been defined as a physician who spends at least 25% of his time in the hospital [22]. However, most hospitalists presently spend far more time in the hospital. The concept of continuous inpatient care in the hospitalist model explains the derivative advantage brought in by hospitalists. Moreover, prior research demonstrated that hospitalists’ value in leading, coordinating and participating in multi-disciplinary teams, to rapidly address and identify acute conditions [23], as well as safely hand off patients [24]. Although patient care outcomes may be maintained by hospitalists, several examinations and key procedures are unavailable during weekends in most institutions [5]. Thus, efficiency of the hospitalists still depends on several non-technical factors in the hospital. In addition, the results of this study indicated that patients had a significantly shorter LOS in the post-intervention group. The complexity of inpatient care and workload of medical personnel may influence the LOS of patients. However, the difference in LOS between weekend and weekday admissions after the implementation of the hospitalist system is still unclear and warrants further research.

Several clinical implications from the study may be used to improve weekend inpatient care. First, this study demonstrates that the implementation of the hospitalist system may provide the same or similar levels of care to weekday- and weekend-admitted patients in the general medicine cohort. Second, there is abundant literature addressing the weekend effect; however, previous studies focus on the phenomenon rather than the solution approach [25]. Not only is it important to note if the weekend effect exists primarily for a certain disease, but it is also mandatory to determine ways to deal with this. For example, adjustment of the weekend staffing model may be valuable for investigating. Third, as is consistent with previous studies, severity and comorbidity tend to be higher in weekend admissions [26–28]. The reasons for these findings were not investigated in the present study. However, a statistical adjustment before making comparison between groups is needed. Moreover, several studies have shown an increased LOS in weekend-admitted patients [5, 28]. Unadjusted data may be not adequately comparable because comorbidity affects mortality, which in turn can affect the LOS. Therefore, a methodology with either a confounder adjustment or matching design should be applied for appropriate comparisons. Forth, researchers may use a variety of quality indicators, rather than mortality alone, to evaluate the weekend effect [21]. It is because with the passage of time, diseases and new illnesses may dilute any effect of the admission time itself [29]. Finally, the effect of the intervention may be sustainable [30, 31] or non-sustainable [32]. Using the data on the implementation of the new hospitalist-care ward over a period of three years showed a sustainable effect on the hospital course and outcome of patients for weekend admissions. The finding implies that redesigning the staffing model during the weekend compared with weekdays could be a sustainable and practical route.

### Strengths and limitations of this study

This study presented both the immediate and long-term intervention effects, and matched the pre- and post-intervention, and the weekday- and weekend-admitted cohort for fair comparisons. In addition, several previous studies on the weekend effect have used administrative data [2, 5, 6], but it has recently been acknowledged that the administrative data was originally collected for non-research purposes [33]. The lack of clinical details, admission route [34] and uncertainty of coding may cause biased results [35]. Although this is a single-institution study, the emergency admission route, detailed clinical course and comparable primary laboratory data may make the study unique, when compared to studies based on secondary analysis.

This study has several limitations. First, it is an observational study with the PSM technique having been done for different characteristics where unmeasured confounders may exist. Although it appears comparable at baseline between the pre- and post-intervention groups, the effect size estimate was still at risk of bias due to residual confounding. We also considered the extent of potential bias from unmeasured and unknown confounders, since the present study did not control for inevitable variations. In addition, of note, PSM may lead to some patients being excluded from the final analyzed sample because they do not have a match within the specified interval on the propensity score. Second, every system has a unique design and instructions for quality control. The root causes of the weekend effect differ depending on the local context [7]. Hence, this single-centre experience cannot be extrapolated to other models and guarantee similar results. Third, it is a controlled before-after study and the patient characteristics might change over time. The new hospitalist-care ward had no controllable pre-intervention cohort because there had been no ward admitting patients exclusively from the ED. That was why we enrolled patients admitted from the ED to several wards with similar case mix in a selected time period, immediately before the hospitalist program, as the comparison cohort. Obviously, our results cannot confirm the earlier evidence on the existence of a persistent weekend effect in other departments of the same hospital or medical institutions. Forth, some outcome events, such as CPR, were extremely rare to tell any difference. We did not overstate that hospitalist system can mitigate these adverse outcomes with extremely low prevalence.

## Conclusions

Apart from a shorter LOS in the post-intervention group, our study revealed no statistically significant differences in hospital mortality, ICU admission and CPR events between weekday and weekend admissions in the pre- and post-intervention groups. In addition, the 7-day hospitalist program may provide equally safe care to weekday- and weekend- admitted patients with sustainable development. This finding suggests that awareness of the weekend effect may have increased deliberate practice and vigilance in the hospital.
